# Lack of evidence for involvement of TonEBP and hyperosmotic stimulus in induction of autophagy in the nucleus pulposus

**DOI:** 10.1038/s41598-017-04876-2

**Published:** 2017-07-03

**Authors:** Chao Liu, Hyowon Choi, Zariel I. Johnson, Jiwei Tian, Irving M. Shapiro, Makarand V. Risbud

**Affiliations:** 1 Department of Orthopaedic Surgery and Graduate Program in Cell and Developmental Biology, Thomas Jefferson University, Philadelphia, PA USA; 2 Department of Orthopaedics, The Central Hospital of Songjiang District, Shanghai, China; 3 Department of Orthopaedics, Shanghai General Hospital, Shanghai Jiao Tong University School of Medicine, Shanghai, China

## Abstract

Nucleus pulposus (NP) cells reside in a physiologically hyperosmotic environment within the intervertebral disc. TonEBP/NFAT5 is an osmo-sensitive transcription factor that controls expression of genes critical for cell survival under hyperosmotic conditions. A recent report on NP and studies of other cell types have shown that hyperosmolarity triggers autophagy. However, little is known whether such autophagy induction occurs through TonEBP. The goal of this study was to investigate the role of TonEBP in hyperosmolarity-dependent autophagy in NP. Loss-of-function studies showed that autophagy in NP cells was not TonEBP-dependent; hyperosmolarity did not upregulate autophagy as previously reported. NP tissue of haploinsufficient TonEBP mice showed normal pattern of LC3 staining. NP cells did not increase LC3-II or LC3-positive puncta under hyperosmotic conditions. Bafilomycin-A1 treatment and tandem mCherry-EGFP-LC3B reporter transfection demonstrated that the autophagic flux was unaffected by hyperosmolarity. Even under serum-free conditions, NP cells did not induce autophagy with increasing osmolarity. Hyperosmolarity did not change the phosphorylation of ULK1 by mTOR and AMPK. An *ex vivo* disc organ culture study supported that extracellular hyperosmolarity plays no role in promoting autophagy in the NP. We conclude that hyperosmolarity does not play a role in autophagy induction in NP cells.

## Introduction

The nucleus pulposus (NP) of the intervertebral disc contains highly hydrated matrix that is primarily composed of large aggregating proteoglycan, aggrecan. The high density of negatively charged sulfated glycosaminoglycans (chondroitin and keratan sulfate) on aggrecan in the confined NP space attract cations and water to provide the tissue with elevated osmotic swelling pressure that resists compressive loading of the spine^1^. Various movements of the spine throughout the day, as well as diurnal loading, lead to dynamic changes of osmolarity within the NP. The baseline osmolarity of NP tissue has been experimentally determined to be in the range of 430–496 mOsm/kg H_2_O^1–4^. Therefore, NP cells reside in a hyperosmotic tissue niche, and have the ability to adapt to the rapid changes in extracellular osmolarity.

TonEBP is a Rel homology transcription factor that controls expression of crucial osmoregulatory genes under hyperosmotic conditions^1, 5, 6^. Our lab has shown that NP cells increase TonEBP in hyperosmotic medium to regulate the levels of transporters and enzymes, such as taurine transporter, betaine-GABA transporter, and aldose reductase, which are critical in maintaining the homeostasis of the intracellular osmolytes and cell volume^7–9^. Importantly, lack of TonEBP under hyperosmotic condition compromises NP cell viability^7^. Thus, NP cells require proper activity of TonEBP for their adaptation and survival in their niche.

Autophagy is a key survival mechanism that can be activated by various stimuli including hypoxia, low nutrient availability, pathogens, and hyperosmolarity^10–13^. When autophagy is activated, cytosolic cargos, such as damaged organelles and misfolded proteins, are encapsulated by double membranous autophagosomes that are tagged by lipid conjugated LC3-II, and subsequently degraded by autophagosome-lysosome fusion^14^. One of the classical regulators of autophagy is MTOR (mechanistic target of rapamycin [serine/threonine kinase]), which serves as an inhibitor of autophagy by phosphorylating ULK1 (unc51-like autophagy activating kinase 1) at Ser757 and disrupting the association between ULK1 and AMPK. Conversely, when MTOR is inhibited, AMPK phosphorylates ULK1 at Ser777, which results in the activation of downstream autophagy related proteins, including BECN1 and ATG12-ATG5^15, 16^.

Hyperosmotic stress has been shown to cause accumulation of inorganic ions, molecular crowding, protein damage and aggregation, as well as DNA damage^17^. In addition, hyperosmotic stress induces autophagy in various cell types and organisms^18–22^. Depending on the context, this induction may serve an osmoprotective role^18, 19, 22^. A recent study in NP cells showed an activation of autophagy by hyperosmolarity through canonical MTOR pathway^23^. Noteworthy, MTOR has been shown to affect TonEBP target expression under hypertonic condition, suggesting a possible crosstalk between autophagic pathway and TonEBP pathway^24^. Since, the relationship between TonEBP and autophagy in NP cells has never been explored, we investigated the role of TonEBP in hyperosmotic induction of autophagy in NP cells. We demonstrate that TonEBP plays no role in controlling autophagic pathway in NP cells, and notably, in contrast to the previous report, our data does not support the conclusion that hyperosmolarity promotes autophagy in NP cells.

## Results

### Autophagy is not regulated by TonEBP in NP cells

**Figure 1:**
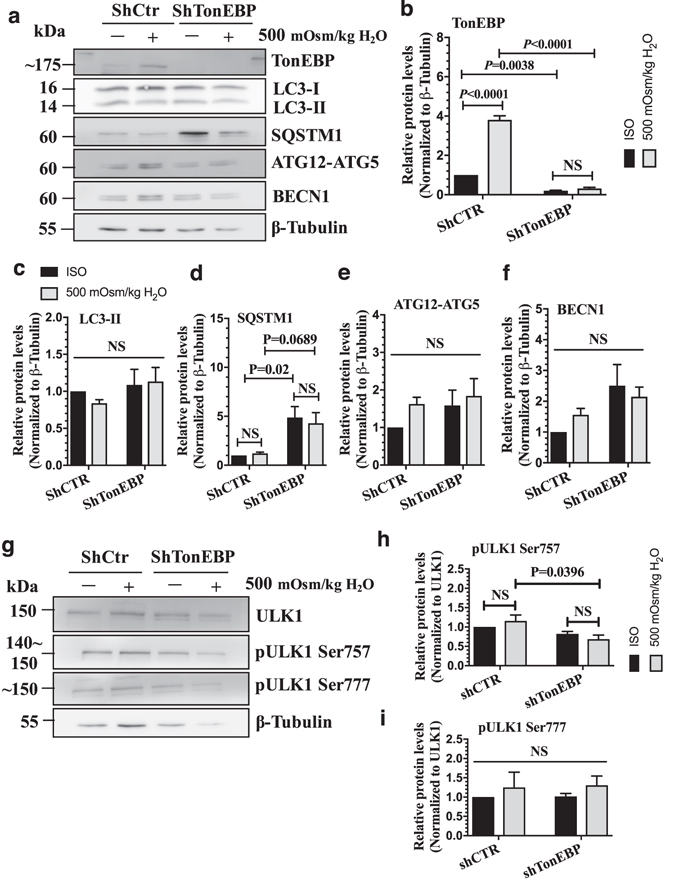
TonEBP does not control autophagy in NP cells. (**a**) Western blot analysis of NP cells transduced with a lentivirus expressing either control shRNA or shTonEBP plasmid showed that TonEBP silencing did not affect the levels of LC3-II, ATG12-ATG5, and BECN1. The levels of SQSTM1 increased with TonEBP silencing. (**b**–**f**) Densitometric analyses of multiple Western blots shown in (**a**). (**g**) Western blot analysis of ULK1 activation status showed that the levels of pULK1 Ser757 was slightly lower, while that of pULK1 Ser777 remained unaffected after TonEBP knockdown under hyperosmotic conditions. (**h**,**i**) Densitometric analyses of multiple western blots shown in (**g**). Bars represent mean ± SEM (n = 4). Two-way ANOVA with Tukey’s multiple comparisons test was used to determine statistical significance. NS, non-significant. Western blot images were cropped and acquired under same experimental conditions. See Supplementary Fig. S1 for examples of uncropped images.

Previous report by Jiang *et al*. showed induction of autophagy in NP cells by hyperosmolarity^23^. Since TonEBP is the major regulator of cellular adaptation to hyperosmotic stress, we asked whether the induction of autophagy under hyperosmotic condition is through TonEBP. Primary NP cells stably transduced with lentivirus expressing either control short-hairpin RNA (shRNA), or shRNA against TonEBP, were cultured under iso- (330 mOsm/kg H_2_O) or hyperosmotic (500 mOsm/kg H_2_O) condition for 12 h, and the level of autophagy was measured. Analysis of Western blots showed that there was a significant (70–80%) decrease in TonEBP levels in TonEBP silenced cells (*P* = 0.0038 for iso-osmotic group; *P* < 0.0001 for 500 mOsm group) and a loss of its induction due to hyperosmolarity (Fig. 1a,b). On the other hand, NP cells transduced with ShCtr upregulated TonEBP levels under hyperosmotic conditions (Fig. 1a,b; *P* < 0.0001). The level of LC3-II, an autophagosome marker, remained unaltered with stable silencing of TonEBP (Fig. 1a,c; n = 4). Surprisingly, we did not observe increased LC3-II levels under the hyperosmotic condition, as reported previously^23^. Moreover, while there was no change in the levels of SQSTM1/p62 with hyperosmotic treatment, a significant increase was observed in TonEBP silenced cells under iso-osmotic (*P* = 0.02) condition, with a similar trend under hyperosmotic (*P* = 0.0689) condition (Fig. 1a,d; n = 4). The levels of other canonical autophagy markers such as ATG12-ATG5 or BECN1 were also not affected by either hyperosmolarity or TonEBP knockdown in NP cells (Fig. 1a,e,f). To delineate if TonEBP had an effect on initiation of autophagy through modulating ULK1 activation status, we measured the phosphorylation of ULK1 at Ser757 by MTOR and at Ser777 by AMPK. Under hyperosmotic condition, phosphorylation of ULK1 at Ser757 was slightly reduced with TonEBP silencing (Fig. 1g,h; *P* = 0.0396; n = 3). However, there was no change in phosphorylation of ULK1 at Ser777 by TonEBP silencing (Fig. 1g,i; n = 3). These results suggested that, while there was some modulation of SQSTM1 levels and ULK1 phosphorlyation at Ser757 by TonEBP, it did not control the canonical autophagic pathway in NP cells.

### Autophagy in NP of TonEBP happloinsufficient mice remains unaltered

**Figure 2:**
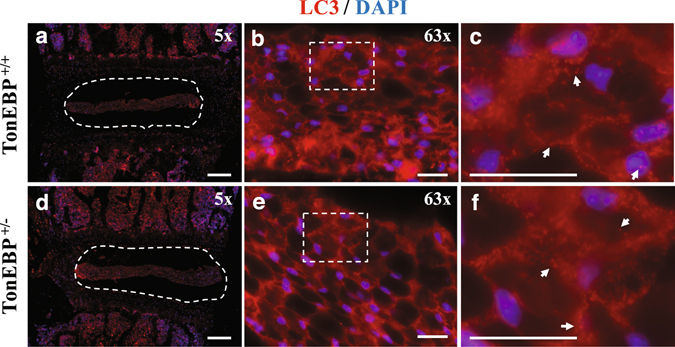
TonEBP haploinsufficient mice do not show altered autophagy in NP. LC3 immunofluorescence staining of intervertebral discs from 4-month-old TonEBP^+/+^ (**a**–**c**) and haploinsufficient TonEBP^+/−^ (**d**–**f**) mice demonstrated similar pattern and distribution of LC3 positive autophagosomes. (**a**,**d**) White dotted line demarks the NP tissue compartment. (**c**,**f**) Magnified images of dotted inserts from B and E respectively. White arrows indicate LC3-positive autophagosomes. Scale bar: 200 μm for (**a**) and (**d**); 20 μm for (**b**), (**c**), (**e**), and (**f**).

To determine if TonEBP affects autophagy *in vivo*, we analyzed the level of LC3 positive puncta in the discs of TonEBP heterozygous mice that are haploinsufficient for TonEBP expression^25, 26^. LC3 immunofluorescence staining showed that the levels and distribution of LC3 positive puncta in the NP were similar in both wild-type and haploinsufficient mice (Fig. 2), suggesting that TonEBP does not control autophagy in NP.

### Levels of autophagy-related proteins do not change in response to hyperosmolarity

**Figure 3:**
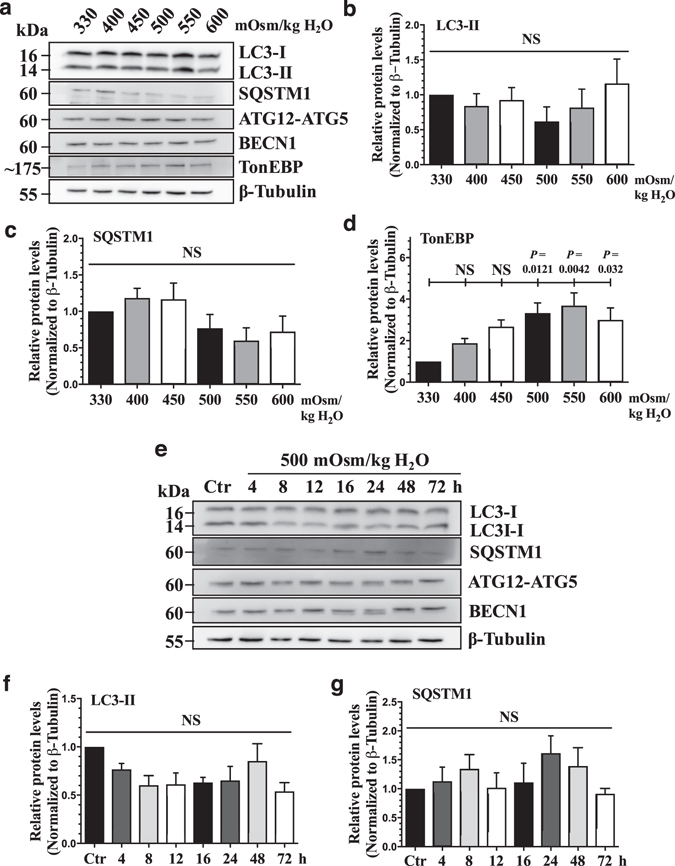
Hyperosmolarity does not upregulate the levels of canonical autophagic markers. (**a**) Western blot analysis of NP cells cultured under increasing osmolarity (330–600 mOsm/kg H_2_O) showed that the levels of LC3-II, SQSTM1, ATG12-ATG5, and BECN1 did not change by hyperosmolarity. However, TonEBP expression increased under hyperosmotic condition. (**b**–**d**) Densitometric analyses of multiple Western blots represented by (**a**) confirmed significant induction of TonEBP, while LC3-II and SQSTM1 levels remained unaltered (n = 5). (**e**) Western blot analysis of NP cells cultured under hyperosmotic condition for increasing lengths of time demonstrated that LC3-II, SQSTM1, ATG12-ATG5, and BECN1 levels were unaffected by hyperosmolarity up till 72 h. (**f**–**i**) Densitometric analyses of multiple Western blots shown in (**e**) (n = 3). Bars represent mean ± SEM. One-way ANOVA with Sidak’s multiple comparisons test was used to determine statistical significance. NS, non-significant. Western blot images were cropped and acquired under same experimental conditions. See Supplementary Fig. S1 for examples of uncropped images.

In contrast to the previous reports, our knockdown studies did not show an evidence of increased autophagy under hyperosmotic condition. While viral transduction has been successfully used to study physiological responses of NP cells to environmental cues^26–29^, to rule out the possibility that viral transduction of primary cells had altered their response to hyperosmotic stimulus, we performed analysis of the autophagic pathway in non-transduced NP cells. NP cells were cultured in media with increasing osmolarity of up to 600 mOsm/kg H_2_O, and the expression levels of autophagic markers were measured. Notably, despite increased osmolarity, Western blots and corresponding densitometric analysis of three independent experiments showed that NP cells were unable to induce levels of LC3-II, SQSTM1, ATG12-ATG5, and BECN1 (Fig. 3a–c; n = 5). On the other hand, as expected, TonEBP was significantly upregulated under hyperosmotic conditions (Fig. 3a,d; *P* = 0.0121 for 500 mOsm/kg H_2_O; *P* = 0.0042 for 550 mOsm/kg H_2_O; *P* = 0.032 for 600 mOsm/kg H_2_O). In addition, to determine if prolonged exposure of NP cells to hyperosmotic conditions promoted autophagy, we cultured the cells under hyperosmotic condition (500 mOsm/kg H_2_O) for varying lengths of time and measured the levels of select autophagy markers (Fig. 3e; n = 3). Under hyperosmotic conditions, NP cells did not change the levels of autophagic markers, LC3-II, SQSTM1, ATG12-ATG5, and BECN1 with time compared to iso-osmotic control (Fig. 3e–g). Taken together, these results ruled out the dose- and time-dependent effect of hyperosmotic stimulus on autophagy marker expression in NP cells.

**Figure 4:**
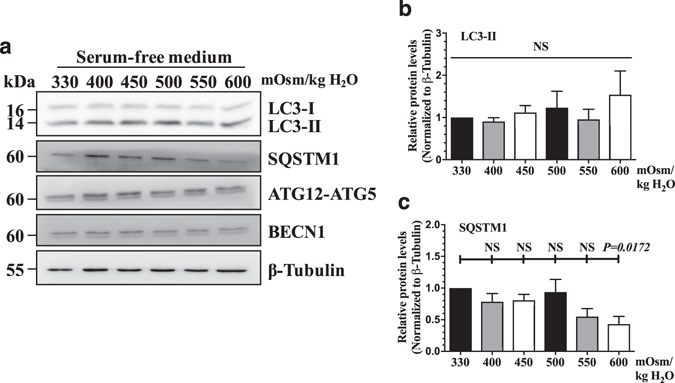
Serum withdrawal does not modulate the effect of hyperosmolarity on autophagy in NP cells. (**a**) Western blot analysis of NP cells cultured in serum-free media with increasing osmolarity (330–600 mOsm/kg H_2_O) showed that the levels of autophagic markers including LC3-II, ATG12-ATG5, and BECN1 were unaltered. SQSTM1 levels did not change with increasing osmolarity except for a small decrease at 600 mOsm/kg H_2_O. (**b**,**c**) Densitometric analyses of multiple Western blots. Bars represent mean ± SEM (n = 5). One-way ANOVA with Sidak’s multiple comparisons test was used to determine statistical significance. NS, non-significant. Western blot images were cropped and acquired under same experimental conditions. See Supplementary Fig. S1 for examples of uncropped images.

Since the NP is avascular, resident cells are thought to have physiologically limited nutrient availability^30, 31^. To better capture the effect of hyperosmolarity on autophagy under these conditions, we cultured NP cells in hyperosmotic medium without serum. Even under serum free conditions, the levels of LC3-II as well as ATG12-ATG5 and BECN1 did not significantly change with hyperosmolarity (Fig. 4a–c). Similarly, there was no change in SQSTM1 levels under hyperosmotic conditions except for a small decrease at 600 mOsm/kg H_2_O that was not accompanied by a corresponding change in LC3-II. These results further suggest that hyperosmolarity is an unlikely autophagy modulator in NP cells (n = 5).

### Autophagic flux is unaffected by hyperosmolarity in NP cells

**Figure 5:**
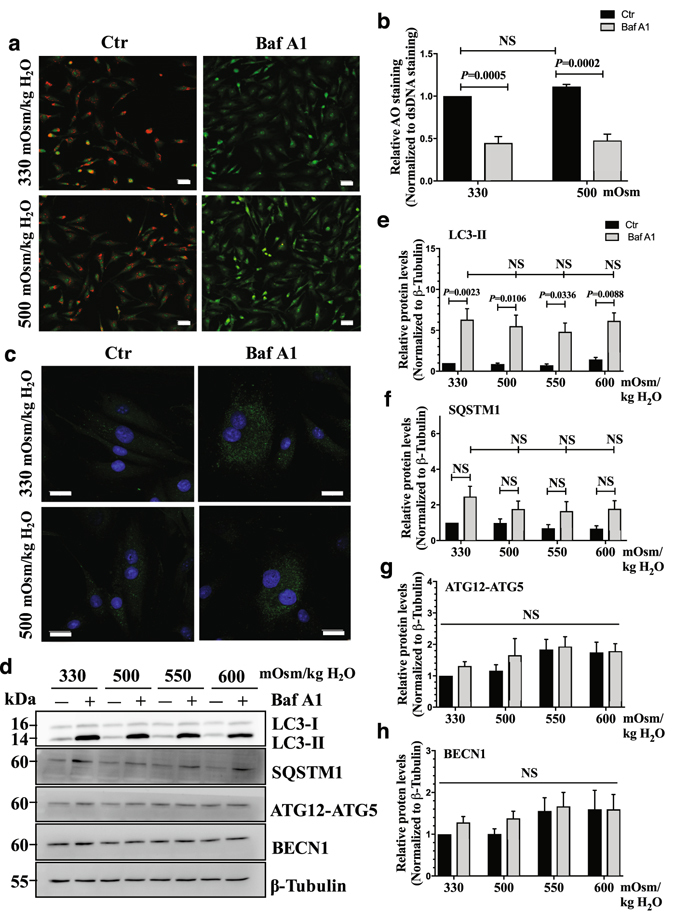
Hyperosmolarity does not influence autophagic flux in NP cells. (**a**) Acridine orange staining of NP cells cultured under iso- (330 mOsm/kg H_2_O, top row) or hyperosmotic (500 mOsm/kg H_2_O, bottom row) condition, treated with (right) or without (left) bafilomycin A1. Hyperosmotic stimulus alone showed no change in number of acidic organelles. Bafilomycin A1 significantly reduced the acridine orange staining irrespective of osmolarity. Scale bar, 35 μm. (**b**) Quantification of acridine orange staining confirmed that hyperosmolarity had no effect on the number of acidic organelles. (**c**) LC3 immunofluorescence staining of NP cells cultured under hyperosmolarity with or without bafilomycin A1 treatment. Hyperosmotic stimulus did not upregulate LC3-positive autophagosomes. Scale bar, 20 μm. (**d**) Western blot analysis of NP cells cultured under increasing osmolarity (330–600 mOsm/kg H_2_O) with or without bafilomycin A1 treatment. The accumulation of LC3-II with bafilomycin A1 treatment was similar under iso- and hyperosmotic conditions. SQSTM1 also showed a trend of accumulation with bafilomycin A1 treatment under all conditions. The levels of ATG12-ATG5 and BECN1 remained unaltered between the experimental groups. (**e**–**h**) Densitometric analyses of multiple Western blots shown in (**d**). Bars represent mean ± SEM (n = 5). Two-way ANOVA with Tukey’s multiple comparisons test was used to determine statistical significance. NS, non-significant. BafA1, bafilomycin A1. Western blot images were cropped and acquired under same experimental conditions. See Supplementary Fig. S1 for examples of uncropped images.

**Figure 6:**
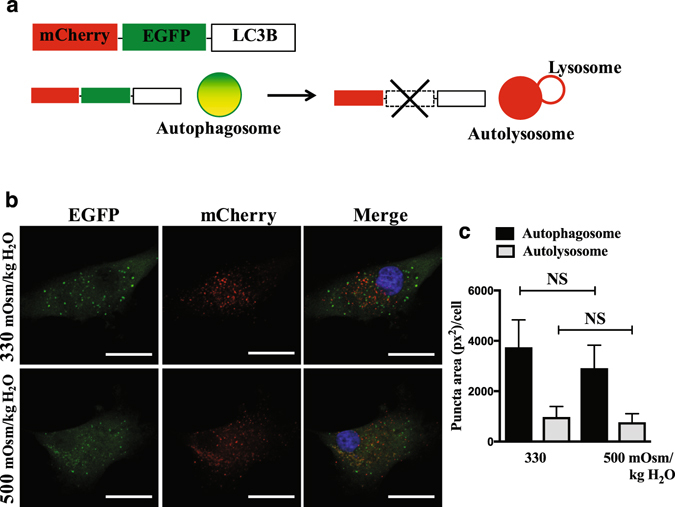
Number of autophagosomes and autolysosomes do not change by hyperosmolarity in NP cells. (**a**) Schematic diagram of tandem mCherry-EGFP-LC3B plasmid. Autophagosomes in the NP cells expressing mCherry-EGFP-LC3B are tagged with both fluorophores and therefore appear yellow/green. When these autophagosomes fuse with lysosomes, acid labile EGFP signal is lost, leaving mCherry signal (red), which measures autophagic flux. (**b**) NP cells transduced with retrovirus expressing a tandem mCherry-EGFP-LC3B construct cultured under either iso- or hyperosmotic condition showed that the numbers of autophagosome (yellow/green puncta) and autolysosome (red puncta) did not change by hyperosmolarity. Scale bar: 25 μm. (**c**) Quantification of puncta area per cell using Colocalization Plugin of ImageJ software confirmed the insensitivity of autophaigc flux to hyperosmotic stimulus. At least 27 cells per group imaged at 126x magnification from 10 random microscopic fields were used for quantification analysis. Bars represent mean ± SEM (n = 3). Student *t* test was used to determine statistical significance. NS, non-significant.

Since hyperosmotic stimulus did not change the levels of autophagic markers, we determined if this was due to its modulation of the autophagic flux. The completion of autophagy involves lysosomal degradation of the autophagosomal contents as well as inner membrane of the autophagosome. Both LC3-II and SQSTM1 are also degraded through this process. Therefore, the accumulation of these proteins or autophagosomes after inhibition of lysosomal degradation reflects the autophagic flux^14^. First, to determine the lysosomal degradation, primary NP cells were cultured under hyperosmotic condition (500 mOsm/kg H_2_O), with or without a lysosomal degradation inhibitor, bafilomycin A1, and then stained with acridine orange to visualize acidic organelles. Hyperosmolarity did not cause increased acridine orange staining in NP cells, indicating that lysosomal degradation was not affected by hyperosmolarity. As expected, bafilomycin A1 significantly reduced the number of acridine orange positive organelles (Fig. 5a,b; *P* = 0.0005 for 330 mOsm/kg H_2_O; *P* = 0.0002 for 500 mOsm/kg H_2_O; n = 3). Similarly, LC3 immunofluorescence staining of NP cells demonstrated that hyperosmolarity did not change the number of LC3-positive autophagosomes (Fig. 5c). Moreover, bafilomyin A1 treatment showed similar accumulation of autophagosomes between iso- and hyper-osmotic condition (Fig. 5c). In addition, we confirmed that increasing osmolarity does not influence autophagic flux in the NP cells. For this purpose, cells were cultured in media with increasing osmolarity (500–600 mOsm/kg H_2_O) with or without bafilomycin A, and the levels of autophagic markers were measured by Western blot (n = 5). Bafilomycin A1 treatment resulted in a significant accumulation of LC3-II under both iso- and hyperosmotic conditions (Fig. 5d,e; *P* = 0.0023 for 330 mOsm/kg H_2_O; *P* = 0.0106 for 500 mOsm/kg H_2_O; *P* = 0.0336 for 550 mOsm/kg H_2_O; *P* = 0.0088 for 600 mOsm/kg H_2_O), indicating that NP cells carryout autophagic degradation as well as do not alter autophagic flux under both iso-osmotic, and hyperosmotic conditions. There was also a consistent trend of accumulation of SQSTM1 under all osmotic conditions, while this did not achieve significance (Fig. 5d,f). This could suggest that SQSTM1 may not be the primary chaperoning protein for shuttling cargos to autophagic degradative pathway in NP cells. Neither ATG12-ATG5 nor BECN1 level was affected by hyperosmolarity or bafilomycin A1 treatment (Fig. 5d,g,h). Furthermore, to better understand the dynamics of autopahgic flux, we stably transduced NP cells with a retrovirus expressing tandem mCherry-EGFP-LC3B construct that marks autophagosomes as green-red (yellow) and autolysosomes as red (Fig. 6a). Transduced cells were cultured under iso- and hyperosmotic conditions, and visualized by fluorescence microscopy (Fig. 6b). Quantification of at least 27 cells per group from three independent experiments indicated that the number of autophagosomes (yellow) and the number of autolysosomes (red) was not significantly different between iso- and hyperosmotic conditions (Fig. 6b,c). Taken together, these results suggest that hyperosmolarity did not significantly affect the autophagic flux in NP cells.

### Hyperosmolarity does not activate ULK1 in NP cells

**Figure 7:**
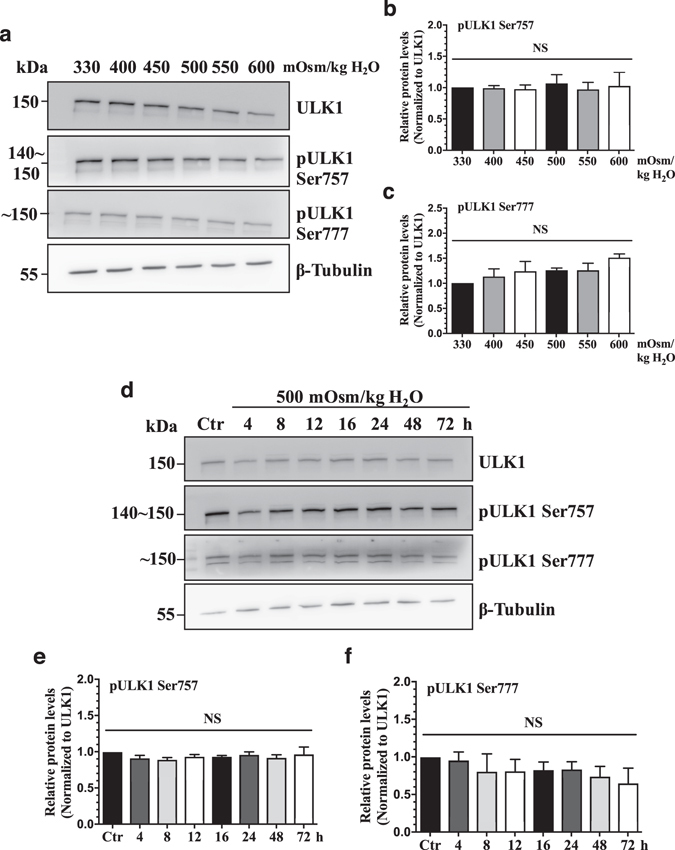
Hyperosmolarity does not activate autophagy through MTOR-AMPK-ULK1 axis in NP cells. (**a**) Western blot analysis of NP cells treated with increasing osmolarity (330–600 mOsm/kg H_2_O) showed that the levels of pULK1 Ser757 and pULK1 Ser777 were not affected by hyperosmolarity. (**b**, **c**) Densitometric analyses of multiple Western blots represented in (**a**) confirmed lack of effect on ULK1 phosphorylaiton at Ser757 and Ser777 by hyperosmolarity. (**d**) Western blot analysis of NP cells cultured in hyperosmotic media for increasing lengths of time demonstrated that phosphorylation of ULK1 at both Ser757 and Ser777 by MTOR and AMPK respectively was not affected by hyperosmolarity till 72 h. (E, F) Denstiometric analyses of multiple Western blots represented in (**d**). Bars represent mean ± SEM (n = 3). One-way ANOVA with Sidak’s multiple comparisons test was used to determine statistical significance. NS, non-significant. Western blot images were cropped and acquired under same experimental conditions. See Supplementary Fig. S1 for examples of uncropped images.

Since autophagic flux was unaffected by hyperosmolarity, we determined if the initiation of autophagy is altered by measuring the levels of p-ULK1 Ser757 and p-ULK1 Ser777. In accordance with the flux data, levels of p-ULK1 Ser757 and p-ULK1 Ser777 in relation to total ULK1 did not change in media with increasing osmolarity (400–600 mOsm/kg H_2_O) (Fig. 7a–c; n = 3). In addition, phosphorylation at either serine residue was not affected by hyperosmotic treatment for up to 72 h (Fig. 7d–f; n = 3), suggesting that hyperosmolarity fails to affect both MTOR and AMPK modulation of ULK1 activity in NP cells.

### Autophagy is not regulated by hyperosmolarity in disc organ culture model

**Figure 8:**
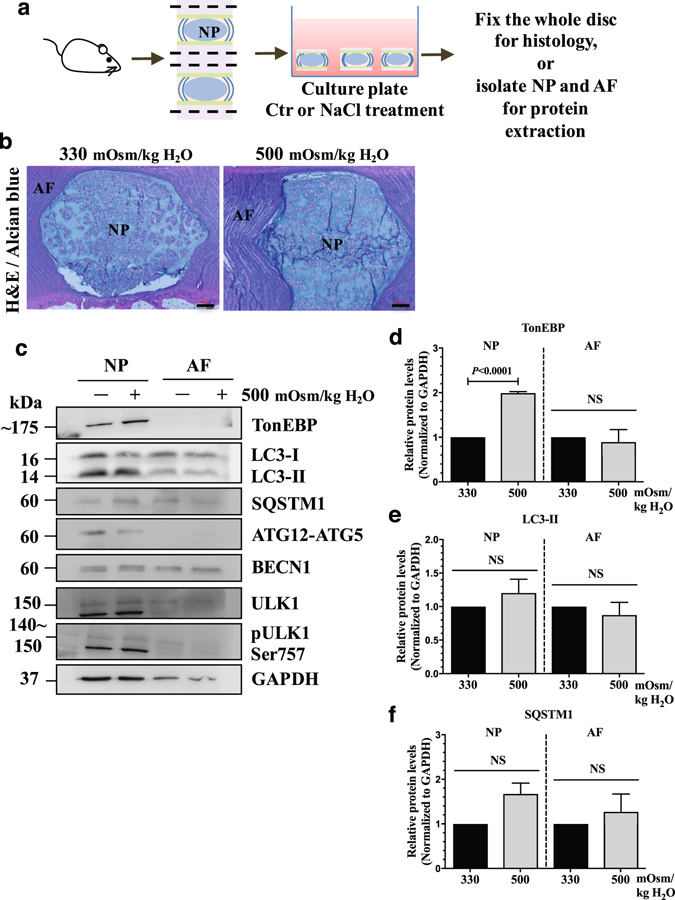
NP cells do not induce autophagy in response to hyperosmotic stimulus in an *ex vivo* disc organ culture model. (**a**) A schematic depicting *ex vivo* rat intervertebral disc organ culture model. (**b**) H&E and alcian blue staining of discs cultured under iso- (330 mOsm/kg H_2_O) or hyperosmotic (500 mOsm/kg H_2_O) conditions showing that NP maintained its structure and cellular morphology. Scale bar: 100 μm. (**c**) Western blot analysis of tissue proteins from NP or AF (annulus fibrosus) compartments of the organ culture discs. The level of TonEBP increased with hyperosmotic stimulus only in the NP. However, the levels of LC3-II, SQSTM1, ATG12-ATG5, BECN1, as well as pULK1 Ser757 did not change with hyperosmolarity in both NP and AF. (**d**–**f**) Densitometric analyses of multiple Western blots represented in (**c**). Bars represent mean ± SEM (n = 3; For each independent experiment, one motion segment per group was used for histology and 6 motion segments per group were used for tissue protein Western blot). Student *t* test was used to determine statistical significance. NS, non-significant. Western blot images were cropped and acquired under same experimental conditions. See Supplementary Fig. S1 for examples of uncropped images.

To further confirm that autophagy in NP is unaltered by hyperosmotic stimulus, we employed rat disc organ culture model where the discs were cultured under either iso- or hyperosmotic condition (Fig. 8a). H&E staining of organ cultured rat discs showed that NP cells and the disc structure were maintained (Fig. 8b). TonEBP was also significantly upregulated in the NP (*P* < 0.0001), but not in the AF in response to hyperosmotic stimulus (Fig. 8c,d; n = 3). Moreover, in both NP and AF, the levels of autophagy markers, LC3-II, SQSTM1, ATG12-ATG5, and BECN1 were similar under both iso- and hyperosmotic conditions (Fig. 8c,e,f). Likewise, neither total ULK1 nor MTOR-mediated phosphorylation of ULK1 at Ser757 was affected by hyperosmolarity in *ex vivo* organ culture of NP and AF (Fig. 8c).

## Discussion

Dynamic fluctuations in extracellular osmolarity can trigger various cellular processes^17^. Osmo-adaptive responses through TonEBP are crucial for maintaining cellular homeostasis and survival under hyperosmotic conditions^1, 9, 32–35^. Autophagy can also serve an osmo-protective role in certain cell types^18, 19, 22^. However, the relationship between TonEBP and autophagy has not been established. Since previous work from our group has shown the importance of TonEBP in NP cell osmo-adaptation^7–9, 26, 33, 34, 36^, and because a recent study reported induction of autophagy in NP cells under hyperosmotic conditions^23^, we investigated the relationship between these pathways. The results of our study clearly showed that autophagy in NP cells is not controlled by TonEBP. Importantly, in contrast to the recent report, we demonstrated that NP cells do not alter autophagic flux in response to hyperosmotic stimulus.

Loss-of-function experiments using lentivirally delivered shRNA targeting TonEBP were performed to establish a relationship between TonEBP and autophagy in NP cells. The results of these silencing experiments that showed no change in levels of LC3-II, ATG12–5, and BECN1, suggested that TonEBP does not control autophagy in NP cells irrespective of extracellular osmolarity. While there was a slight decrease in the level of p-ULK1 Ser757 with TonEBP knockdown under hyperosmotic condition, absence of increase in autophagy activating phosphorylation of ULK1 at Ser777 indicated that autophagy was not induced. Interestingly, the observation that SQSTM1 was increased by TonEBP knockdown without concomitant change in LC3-II levels suggests that the alteration in SQSTM1 is not due to modulation of autophagic flux. Alternatively, TonEBP may control SQSTM1 expression, a possibility supported by our recent RNA-sequencing study (GSE86552)^26^. Further evidence supporting the lack of involvement of TonEBP in modulating autophagy was the observation that NP tissue in TonEBP haploinsufficient mice do not show altered level and pattern of LC3-positive autophagosomes.

Surprisingly, the TonEBP loss-of-function studies did not show induction of autophagy in NP cells in response to hyperosmotic stimulus. To rule out the possibility that viral transduction has altered cellular response to increase in osmolarity, we conducted a series of experiments using non-transduced NP cells. The results of time- and dose- response experiments of hyperosmolarity confirmed that hyperosmotic stimulus does not affect the levels of autophagic markers. A similar observation was made when hyperosmotic stimulus was combined with serum-free condition as an additional stressor and to mimic nutrition-limited conditions *in vivo*. Both of these results suggested that the levels of autophagic markers are insensitive to increases in extracellular osmolarity. It is plausible that absence of LC3-II induction could be due to simultaneous increase in the autophagic flux. However, the results of the experiments that measure the accumulation of LC3-II following bafilomycin A1 treatment did not support this hypothesis. In addition, similar levels of autophagosomes and autolysosomes were observed under both iso- and hypertonic conditions in NP cells transduced with tandem mCherry-EGFP-LC3B construct, further suggesting that hyperosmolarity does not affect autophagic flux in NP cells. Our data also showed that both MTOR and AMPK activity on autophagy modulation did not change under hyperosmotic condition. It is true that NP cells increase intracellular calcium in response to hyperosmolarity^9^, but our results indicate that it does not result in increased AMPK phosphorylation of ULK1 at Ser777 to initiate autophagy. Similarly, MTOR phosphorylation of ULK1 at Ser757 does not change under hyperosmolarity. It is evident that hyperosmolarity does not affect the initiation of autophagy in NP cells. Furthermore, our *ex vivo* organ culture study, where NP cells are maintained in their native extracellular niche, strongly supports the findings from cell culture studies that NP cells do not induce autophagy in response to increased osmolarity.

Taken together, our results contradict the findings by Jiang *et al*. that showed increased autophagic flux by hyperosmotic stimulus^23^. This discrepancy could stem from multiple sources. Most importantly, the transmission electron microscope images of NP cells shown by Jiang and colleagues show that the size of the cells used in their experiments is approximately 12 μm. This is significantly smaller than the reported size of rat NP cells (24.5 ± 7.6 μm)^37, 38^. Moreover, NP cells are reported to have very few mitochondria^39, 40^, a stark contrast to the cells used by Jiang *et al*. which show numerous well-organized mitochondria. These observations suggest that the cells used for the analysis were contaminated with cells from another tissue source. In addition, it has been previously shown that NP cells have active autophagic flux under basal conditions^38^, which again disagrees with the report demonstrating autophagic flux occurring only when cells were cultured under hyperosmolarity. This further indicates that the cells used by Jiang *et al*. were not purely derived from the NP. Moreover, although Jiang *et al*.^23^ have shown that phosphorylation of P70S6K is decreased by hyperosmotic stimulus, they did not measure the MTOR activity on autophagic pathway, namely, ULK1 phosphorylation at Ser757, which our studies clearly showed remained unaffected. In conclusion, our results suggest that increases in extracellular osmolarity and TonEBP do not play a role in controlling autophagy in NP cells.

## Materials and Methods

### Reagents and Plasmids

Lentiviral ShTonEBP (TRCN0000020019) and control ShRNA plasmids were purchased from Sigma. pBABE-puro mCherry-EGFP-LC3B (22418) developed by Dr. Jayanta Debnath^41^, and psPAX2 (12260) and pMD2.G (12259) developed by Dr. Didier Trono were obtained from Addgene.

### Cell culture and treatments

All procedures regarding collection of animal tissues was performed as per approved protocols by Institutional Animal Care and Use Committee (IACUC) of the Thomas Jefferson University, in accordance with the IACUC’s relevant guidelines and regulations. Rat NP cells were isolated using a method described by Risbud *et al*.^42^ After isolation, cells were maintained in Dulbecco’s Modified Eagles Medium (DMEM) (Corning, 10–013-CV) with 10% fetal bovine serum (FBS) (Sigma-Aldrich, F6178) supplemented with antibiotics in T25 flask until confluent (P0), and then passaged into bigger flask for expansion (P1). Cells up to P4 were used for the experiments. For hyperosmotic treatment, NP cells were cultured in normoxia (20.9% pO_2_) in 1 g/L glucose DMEM with 10% FBS, containing either no additional NaCl (330 mOsm/kg H_2_O) or additional NaCl (400–600 mOsm/kg H_2_O) for 4–72 h. Based on the manufacturer’s given DMEM osmolality, appropriate amount of NaCl was added to make hyperosmotic medium. In some experiments, cells were pre-treated with rapamycin (EMD Millipore, 553210) (100 nM) 1 h prior to NaCl treatment. To inhibit autophagic flux, cells were treated with bafilomycin A1 (Tocris, 1334) (50 nM) for the last 2 h of NaCl treatment.

### Acridine Orange Staining

NP cells were plated in 24-well plates, and cultured in control (330 mOsm/kg H_2_O) or hyperosmotic medium (500 mOsm/kg H_2_O) with or without rapamycin or bafilomycin A1. At the last 30 min before completing hyperosmolarity treatment, acridine orange (Sigma Aldrich, A9231) was added at 1 μg/mL concentration. After 30 min incubation in dark, media was replaced with PBS (Corning, 46–013-CM), and the cell images were taken using Zeiss Axio Imager.A2 microscope (Carl Zeiss, Germany), or the fluorescence intensity was measured at 488/525 nm (excitation/emission) for DNA bound green signal or at 488/650 nm for acidic red signal using Infinite® M1000 Pro microplate reader (Tecan, Switzerland). Three independent experiments were performed with three technical replicates per experiment. Quantification of acridine orange staining was calculated by normalizing fluorescence readings at 650 nm to that of 525 nm.

### Tandem mCherry-EGFP-LC3 immunofluorescence

Phoenix-AMPHO cells (ATCC, CRL-3213) were plated in 10 cm plates (5 × 10^6^ cells/plate) in DMEM with 10% heat-inactivated FBS one day before transfection. Cells were transfected with 20 μg of pBABE-puro-mCherry-EGFP-LC3B. Lentiviral particles were harvested at 48 to 60 h post-transfection. NP cells were plated on glass coverslips in DMEM with 10% heat-inactivated FBS one day before transduction. Cells were transduced with virus medium along with 8 μg/ml polybrene (Sigma Aldrich, H9268). 24 h later, the medium was replaced with 1 g/L glucose DMEM (Gibco, 11885084) with 10% FBS, and the cells were cultured in 500 mOsm/kg H_2_O for 12 h. After the treatment, cells on the coverslips were fixed with 1% paraformaldehyde (PFA) (Sigma-Aldrich, 158127) in PBS for 15 min at room temperature in dark, washed with PBS thoroughly, and mounted with ProLong® Gold Antifade Mountant with DAPI (Thermo Fisher Scientific, P36934) for viewing under the microscope. Images of multiple cells from three independent experiments were taken using Zeiss LSM510 confocal microscope (Carl Zeiss, Germany). Quantification of green/yellow and red only puncta based on their co-localization was measured as their area (pixel^2^/cell) using Colocalization Plugin of ImageJ software (http://rsb.info.nih.gov/ij/). At least 27 cells per group from three independent experiments were analyzed.

### Immunofluorescence microscopy

TonEBP^+/+^ and haploinsufficient TonEBP^+/−^ mice generated by Dr. Steffan N. Ho were a kind gift from H. Moo Kwon, Ulsan National Institute of Science and Technology, Ulsan, Republic of Korea^25^. Four-month-old mice were sacrificed according to the approved protocols and guidelines set forth by the Thomas Jefferson University’s IACUC. Mouse lumbar spine tissues were immediately fixed in 4% PFA in PBS at 4 °C for 48 h, decalcified with 20% EDTA at 4 °C for 15 days, and then embedded in paraffin. Sagittal sections of 6–8 μm thickness were cut. For localizing LC3, sections were de-paraffinized and incubated in microwaved citrate buffer for 20 min for antigen retrieval. Then the sections were blocked in 5% normal goat serum (Thermo Fisher Scientific, 10000 C) in PBS-T (0.4% Triton X-100 in PBS), and incubated with anti-LC3 antibody (Novus Biologicals, NB100-2220) in 5% normal goat serum in PBS-T (0.4% Triton X-100 in PBS) at a dilution of 1:200 at 4 °C overnight. Tissue sections were thoroughly washed and incubated with Alexa Fluor®-594 conjugated anti-rabbit secondary antibody (Jackson ImmunoResearch Lab, Inc., 711-586-152), at a dilution of 1:800 for 1 h at room temperature in dark. The sections were washed again with PBS-T (0.4% Triton X-100 in PBS) and mounted with ProLong® Gold Antifade Mountant with DAPI (Thermo Fisher Scientific, P36934).

For cultured cell immunofluorescence staining, NP cells were plated on glass coverslips. After treatments, cells were fixed and permeabilized with cold methanol at −20 °C for 15 minutes, washed with PBS and then blocked with 5% normal goat serum in PBS with 0.3% Triton X-100 (Sigma Aldrich, T8787) for 1 h at room temperature. Cells on coverslip were then incubated with anti-LC3 antibody (Cell Signaling Technology, 12741) in blocking buffer at a dilution of 1:100 at 4 °C overnight, washed with PBS, and then incubated with Alexa Fluor®−488 conjugated anti-rabbit secondary antibody (Jackson ImmunoResearch Lab, Inc., 711-545-152), at a dilution of 1:800 for 1 h at room temperature in dark. Then the coverslips were washed with PBS and mounted with ProLong® Gold Antifade Mountant with DAPI (Thermo Fisher Scientific, P36934). All mounted slides were visualized using a Zeiss AxioImager A2 (Carl Zeiss, Germany). Three independent experiments were performed.

### Protein extraction and Western blotting

Following treatment, cells were immediately placed on ice and washed with ice-cold PBS. All the wash buffers and the final cell lysis/re-suspension buffers included 1X cOmplete^TM^ Mini Protease Inhibitor Cocktail (Roche, 11836153001), NaF (5 mM) (Sigma Aldrich, 201154) and Na_3_VO_4_ (200 μM) (Sigma Aldrich, S6508). Total cell proteins were resolved by electrophoresis on 8–12% SDS-polyacrylamide gels and transferred by electroblotting to PVDF membranes (EMD Millipore, IPVH00010). The membranes were blocked with 5% non-fat dry milk in TBST (1% Tween 20 in TBS) and incubated overnight at 4 °C in 5% non-fat dry milk in TBST with the antibodies against LC3 (1:1000, 12741), BECN1 (1:1000, 3495), SQSTM1/p62 (1:1000, 5114), ATG12 (1:1000, 4180), pULK1 Ser757 (1:1000, 14202), or ULK1 (1:1000, 8054) (Cell Signaling Technology). The membranes were also incubated with primary antibodies against, TonEBP/NFAT5 (1:1000, Novus Biologicals, NB120–3446), pULK1 Ser777 (1:500, EMD Millipore, ABC213), or β-Tubulin (1:10,000, DSHB, E7). Immunolabeling was detected using the Amersham^TM^ ECL^TM^ Prime Western Blotting Detection Reagent (Thermo Fisher Scientific, 45–002–401). All Western blot experiments were performed three independent times.

### Lentiviral particle production and viral transduction

HEK 293 T cells (ATCC, CRL-3216) were plated in 10 cm plates (5 × 10^6^ cells/plate) in DMEM with 10% heat-inactivated FBS one day before transfection. Cells were transfected with 9 μg of ShCtr or ShTonEBP plasmids along with 6 μg psPAX2 and 3 μg pMD2.G using Lipofectamine 2000 (Invitrogen). After 6 h, transfection medium was replaced with DMEM with 10% heat-inactivated FBS and penicillin-streptomycin. Lentiviral medium was harvested at 48 to 60 h post-transfection, and mixed with 7% PEG 6000 (Sigma Aldrich, 81253) solution and incubated overnight at 4 °C to precipitate virus particles. PEG solution was removed from virus medium before transduction by centrifugation at 1,500 × *g* for 30 min to pellet virus particles. NP cells were plated in DMEM with 10% heat-inactivated FBS one day before transduction. Cells were transduced with fresh DMEM with 10% heat-inactivated FBS containing viral particles along with 8 μg/ml polybrene (Sigma Aldrich, H9268). 16 h later, the medium was removed and replaced with DMEM with 10% FBS. Cells were harvested for protein extraction 4–5 days after transduction to ensure maximum knockdown efficiency without affecting cell viability. Three independent experiments were performed.

### *Ex Vivo* disc organ culture

Rats were sacrificed according to the approved protocols and guidelines set forth by the Thomas Jefferson University’s IACUC. Whole spines were dissected from rat *en-bloc* and muscle, tendon, and ligaments were carefully removed. Individual motion segments of intervertebral disc were cut close to the end plate with a scalpel and allowed to equilibrate overnight in DMEM with 10% FBS supplemented with antibiotics. Seven motion segments per group were used. The next day, the motion segments were cultured in either control or 500 mOsm/kg H_2_O medium for 8 h. After the treatment, one motion segment from each group was fixed in 4% PFA for 48 h, and then decalcified with 20% EDTA for paraffin embedding. 7 μm sections were cut and stained with H&E and alcian blue. Six motion segments per group were further dissected to isolate NP and AF tissue proteins. Three independent experiments were performed.

### Statistical analysis

All experiments were performed at least three independent times. Data is presented as the mean ± SE. Differences between multiple groups were assessed by either one-way or two-way ANOVA depending on the number of variables with appropriate post-hoc analyses (Sidak’s and Tukey’s multiple comparisons test), and the differences between two groups were assessed by Student *t* test using Prism7 (GraphPad Software). *P* < 0.05 was considered statistically significant.

## Electronic supplementary material

Supplementary Information
