# Supplementary material for: Lack of evidence for involvement of TonEBP and hyperosmotic stimulus in induction of autophagy in the nucleus pulposus
Source: Sci Rep. 2017 Jul 3;7:4543. doi: 10.1038/s41598-017-04876-2 (PMC5495809; doi:10.1038/s41598-017-04876-2)
Supplement: Supplementary file 1 — Supplementary Information [file 41598_2017_4876_MOESM1_ESM.pdf]

Supplementary Information

Lack of evidence for involvement of TonEBP and hyperosmotic stimulus in induction of autophagy in the nucleus pulposus

Chao Liu[1,2], Hyowon Choi[1], Zariel I. Johnson[1], Jiwei Tian[2], Irving M. Shapiro[1], Makarand V. Risbud[1*]

Supplementary Figure S1-1. Examples of uncropped Western blots

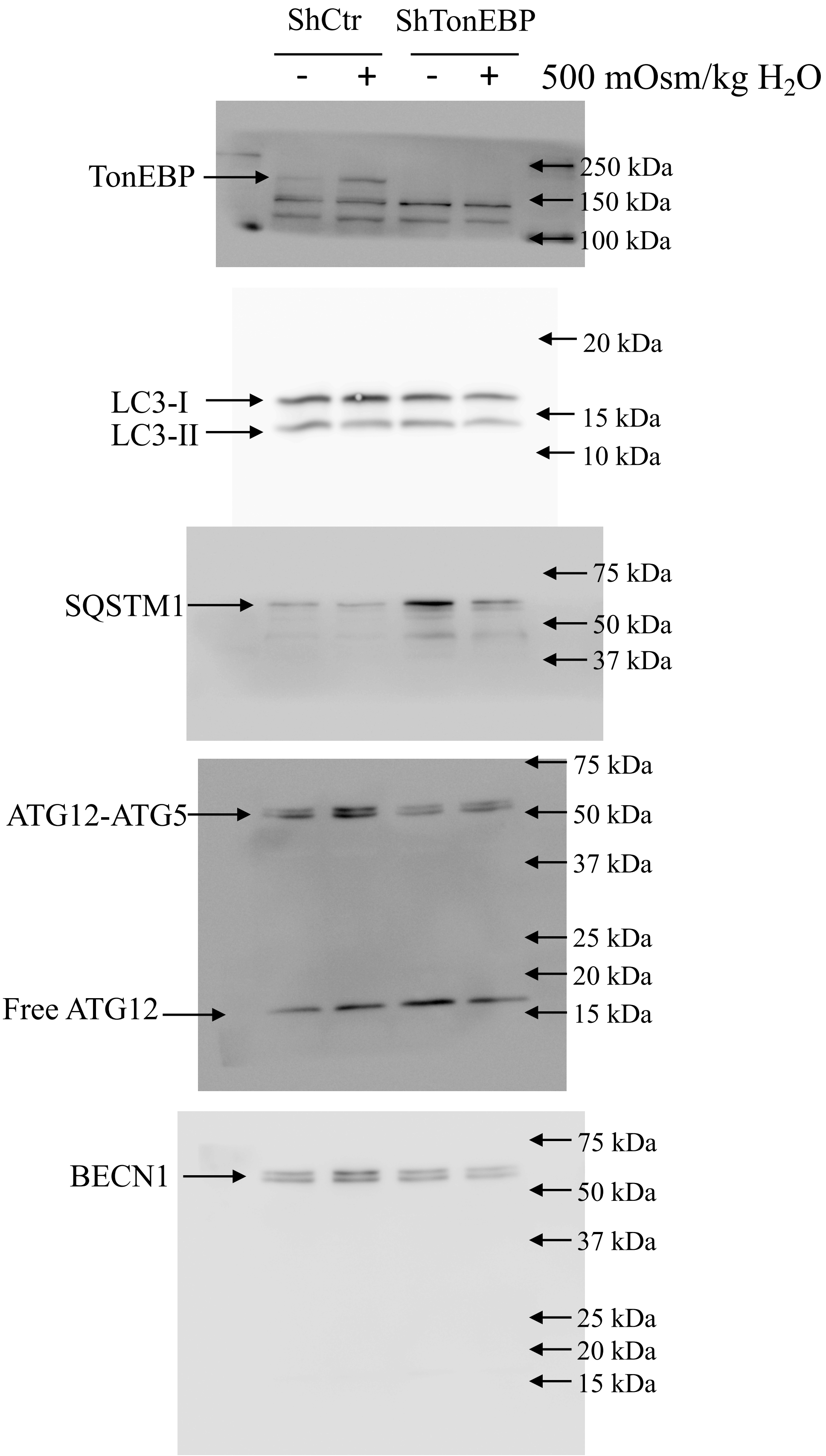

Examples of uncropped western blots for TonEBP, LC3, SQSTM1, ATG12-ATG5, and BECN1 from Figure 1A

Supplementary Figure S1-2. Examples of uncropped Western blots

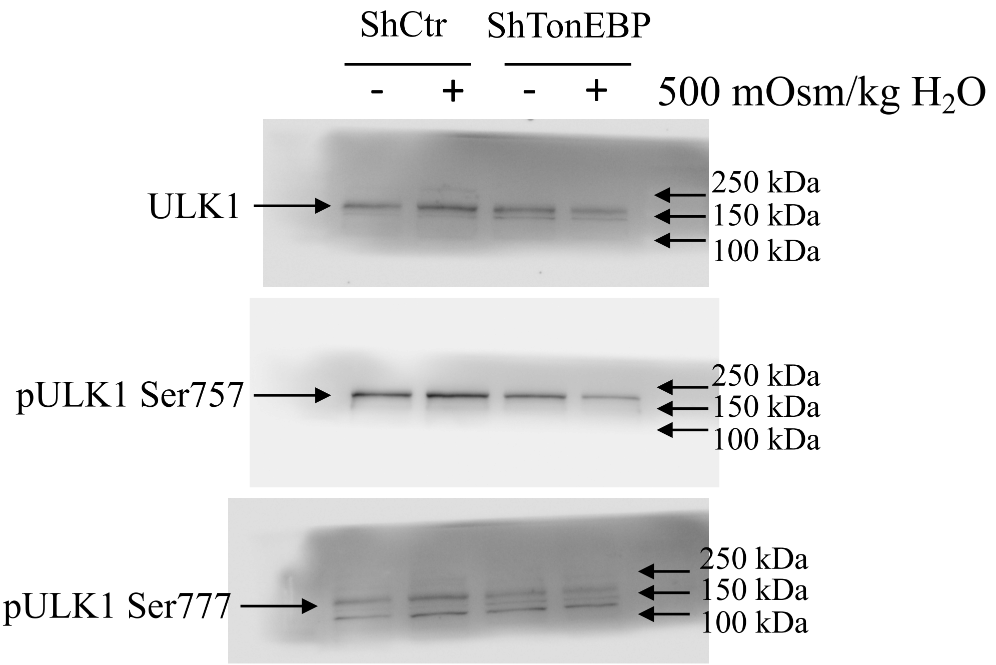

Examples of uncropped western blots for ULK1, pULK Ser757, and pULK Ser777 from Figure 1G

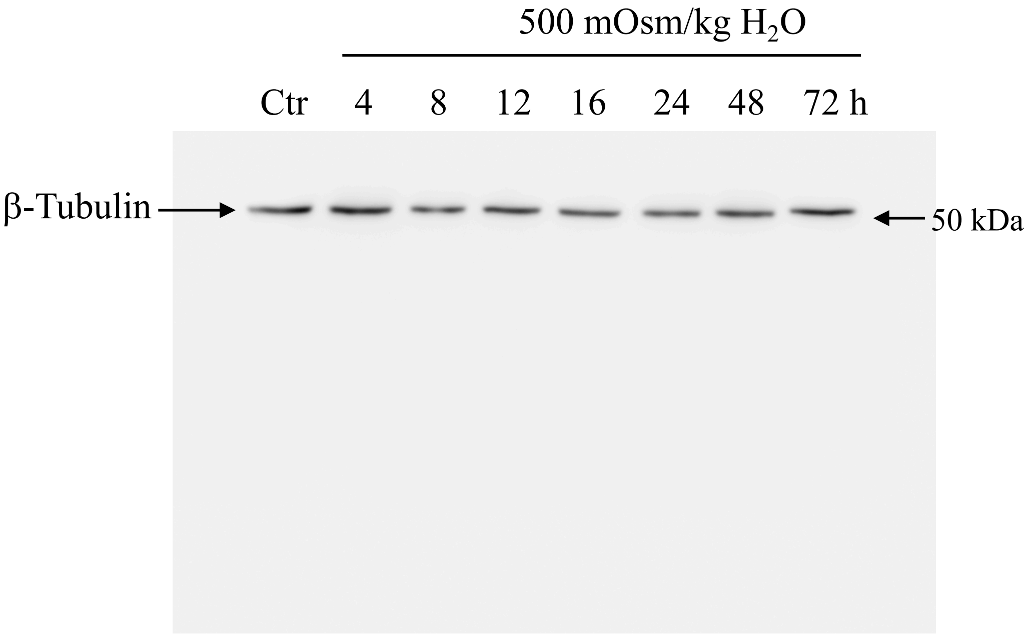

Example of uncropped western blot for β-Tubulin from Figure 3E

Supplementary Figure S1-3. Example of uncropped Western blot

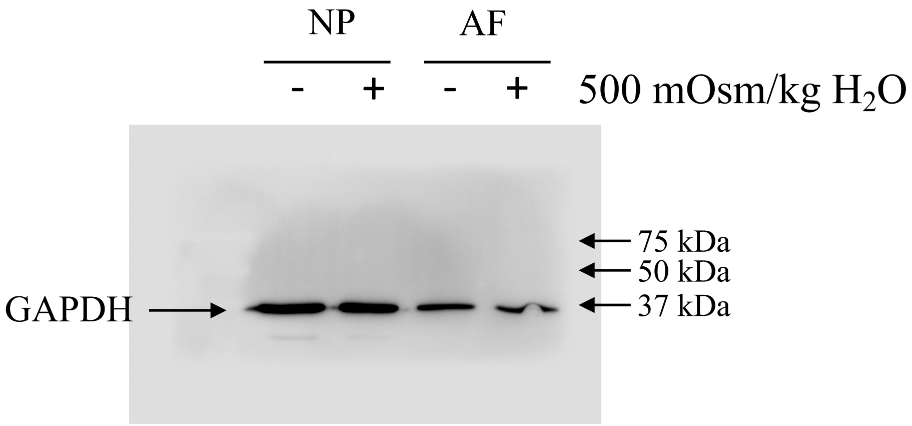

Example of uncropped western blot for GAPDH from Figure 8C
